# Clinicopathological characteristics and clinical outcomes of multiple histological subtypes in feline mammary carcinomas

**DOI:** 10.1177/03009858261462564

**Published:** 2026-07-13

**Authors:** Pruettha Aruvornlop, Nlin Arya, Sirintra Sirivisoot, Tanit Kasantikul, Rassameepen Phonarknguen, Walasinee Sakcamduang

**Affiliations:** 1 Faculty of Veterinary Science, Mahidol University, Nakhon Pathom, Thailand; 2 Faculty of Veterinary Science, Chulalongkorn University, Bangkok, Thailand; 3 Veterinary Diagnostic Laboratory, Michigan State University, East Lansing, MI

**Keywords:** feline, histological types, mammary carcinoma, prognosis, survival analysis

## Abstract

Feline mammary carcinomas (FMCs) are heterogeneous neoplasms often comprising multiple histological patterns within a single case. The current practice of focusing on a prominent subtype hinders prognostic accuracy and potentially produces suboptimal therapeutic decisions. This retrospective study proposed criteria for grouping these mixed patterns and evaluated their prognostic significance in FMCs. The criteria were based on the tubular carcinoma composition and adopted from those of human breast cancer. Eighty-seven cats with FMCs were divided into groups according to tubular carcinoma composition: pure tubular carcinoma (PTC, *n* = 34), tubular carcinoma mixed with other subtypes (TMC, *n* = 16), and nontubular carcinoma (NTC, *n* = 37). Prognostic factors and overall survival (OS) were compared among the groups. The PTC and TMC groups were significantly associated with early-stage FMCs (*P* = .03) and low argyrophilic nucleolar organizer region values (*P* = .02). Conversely, NTCs had the poorest prognosis (*P* = .03) and were associated with incomplete surgical margins (*P* = .004), advanced clinical stage (*P* < .001), and high histological grade (*P* < .001) on multivariable analysis. Median OS for the PTC, TMC, and NTC groups was 221, 242, and 147 days, respectively. Considering individual histological subtypes, cats with adenosquamous carcinoma had a shorter OS (60 days) than those with comedocarcinoma (147 days) and tubular carcinoma (240 days, *P* < .001). These criteria were associated with FMC outcome in this cohort, providing a potentially useful framework for prognosis. However, further validation through prospective studies is needed to fully establish their clinical utility.

Feline mammary carcinomas (FMCs) are the most common type of neoplasia arising from the epithelium of the mammary gland. This malignancy is highly aggressive and associated with a poor prognosis.^1,4^ FMCs are classified into various subtypes based on their distinct histological patterns, such as tubular carcinoma, solid carcinoma, comedocarcinoma, and micropapillary carcinoma. In canine mammary neoplasia, histological classification and grading are crucial for determining prognostic parameters.^8,19,28^ However, the prognostic significance of histological classification in FMCs is complicated by inconsistencies in the literature. While some studies have linked survival to specific histological subtypes, including solid, micropapillary, tubulopapillary, and complex carcinomas (currently classified as nonsimple carcinomas),^14,23–25^ others have found no such correlations.^2,4,13,30^ Moreover, the prognostic value of certain histological subtypes in FMCs, such as comedocarcinoma and adenosquamous carcinoma, remains critically underexplored.^21^

The histopathologic classification of FMCs can be challenging. The microscopic characteristics of samples need to be evaluated by experienced pathologists to determine the subtype(s) and tumor grade. The multicentricity of tumors further complicates this process, as a cat can develop more than 1 neoplastic subtype, and individual tumors often exhibit the features of several different subtypes.^16,23,33^ Most studies on both canine and feline mammary tumors have addressed the presence of multiple histological patterns by relying on the most prominent growth pattern for final subclassification.^6,14,27^ However, this practice raises a crucial question regarding prognostic accuracy: Does evaluating only the dominant subtype truly represent the patient’s full prognostic risk? Given the limited research evaluating the prognostic impact of multiple coexisting subtypes, the omission of less dominant yet potentially aggressive subtypes may underestimate the true prognostic risk.^11^

Tubular and tubulopapillary carcinomas are the most common FMC subtypes,^35^ which often coexist with other histological subtypes, especially solid carcinoma and comedocarcinoma.^34^ A recent review emphasized the lack of standardized rules or established criteria for classifying tumors with multiple histological patterns in FMCs, which can cause diagnostic ambiguity and complicate prognostic assessments.^21^

The challenges in classifying multiple histological subtypes of mammary tumors in veterinary medicine mirror those in human oncology. In human breast cancer, a mixture of tubular carcinoma and other histological subtypes can significantly affect tumor characteristics and influence prognoses. The nontubular component in these mixtures affects lymph node metastasis, molecular classification, treatment, and overall prognosis of breast cancer.^31,36^ Given this established precedent in human pathology and the histopathological similarities between human and feline mammary cancer,^10^ adopting classification criteria based on human breast cancer may help establish a crucial comparative oncology framework. This approach facilitates an investigation into the impact of a nontubular component on prognostic risk in FMCs, thereby helping improve the knowledge base of feline mammary oncology.

This retrospective study sought to propose criteria for grouping multiple mammary carcinomas based on the presence of tubular carcinoma, ie, categorizing cases into pure tubular carcinoma (PTC), tubular mixed carcinoma (TMC), or nontubular carcinoma (NTC). Furthermore, we also evaluated the prognostic value of histological subtypes, including both predominant and multiple histological subtypes, in FMCs by comparing clinicopathological prognostic factors, proliferative markers, and clinical outcomes.

## Materials and Methods

### Case Selection

This retrospective study analyzed clinical and pathological records retrieved from the medical archives of the Veterinary Diagnostic Center, Mahidol University, Thailand, between 2014 and 2022. The protocol for animal use in this study was approved by Mahidol University–Institute Animal Care and Use Committee of the Faculty of Veterinary Science (MUVS 2020-08-39 and MUVS 2021-02-04). The cohort consisted of 87 female cats diagnosed with FMC. All subjects underwent a complete physical examination, thoracic radiography to screen for distant metastasis, and surgical resection with or without subsequent adjuvant chemotherapy. Inclusion in the cohort was independent of tumor size, vascular invasion, peritumoral infiltration, or lymph node metastasis status. Cats with other concurrent malignant tumors, those that received chemotherapy before surgical resection, and those diagnosed based on incisional biopsy alone were excluded.

### Data Collection

In total, 103 individual tumor masses (accounting for both single and multiple tumors per cat) were obtained and analyzed. Clinical data on gross lesions were obtained from medical records, including number of lesions, anatomical location of tumors, type of surgery, and treatment regimen. The tumor diameter was measured using the metric system upon surgical resection, prior to fixation. When available, lymph node status confirmed by cytology was recorded. In cases with multiple tumors, the size of the largest tumor was used to represent the overall tumor size.

All cases were clinically staged using a modified World Health Organization staging system.^15^ The stages were defined as: stage I (tumor size <2 cm, no evidence of metastasis), stage II (tumor size 2–3 cm, no metastasis), stage III (tumor size >3 cm or regional lymph node metastasis), and stage IV (any tumor size with evidence of distant metastasis).

### Histology and Grading

In total, 103 individual tumor masses from 87 cats were histologically analyzed. In cats with multiple masses, all lesions submitted by the surgeon (typically 1–3 masses per cat) were examined histologically. The masses collectively constituted the representative tumor for that individual cat. All samples of mammary masses and lymph nodes were embedded in paraffin blocks, which were then cut into 5-µm-thick sections and stained with hematoxylin and eosin. Microscopic analyses of samples were performed jointly by TK, SS, NA, and PA.

Neoplasms were graded using the grading system established by Mills et al.^14^ Briefly, 3 tissue histological characteristics were assessed: nuclear form, lymphovascular invasion (LVI), and mitotic count (MC). Each histological feature was assigned a score of 0 or 1. Nuclear form was assigned a score of 0 if ≤5% of nuclei exhibited abnormal shapes (eg, corrugation, angularity, clefting, or indentation) and a score of 1 if >5% of nuclei were abnormal. MC was assessed in 10 consecutive high-power fields (HPFs; 0.55 mm field diameter; total area of 2.37 mm^2^ at 400× magnification; CARL ZEISS: AXIOSKOP 40FL, Oberkochen, Baden-Württemberg, Germany). A score of 0 was assigned if ≤62 mitoses per 10 HPFs, and a score of 1 was assigned if >62 mitoses per 10 HPFs. The total score (0–3) for these parameters determined the histological grade: 0 = grade 1; 1 = grade 2; and 2–3 = grade 3. In cats with multiple masses, the mass with the highest score determined the overall grade. Surgical margins were evaluated using cross-sectional and longitudinal tumor sections. A margin was complete if neoplastic cells were >2 mm from the closest margin and incomplete if the distance was ≤2 mm.^7^

### Quantification of Histological Subtyping

Histological subtypes were determined by histological subtyping and histological grouping. The histological subtype of each cat was defined as the predominant morphologic pattern constituting ≥50% of the of the total viable tumor area, as determined using the international histological classification for feline mammary tumors.^34^ To establish the viable tumor area, ImageJ software (National Institutes of Health, Bethesda, Maryland) was used to delineate and manually exclude nonmalignant components (ie, necrosis, fibrosis, and inflammation) from the total area. Thus, the remaining area, ie, the viable malignant area, constituted 100% of the base for subsequent subtype quantification.

Intratumoral (variation within a single mass) and intertumoral (variation between different masses) heterogeneity were characterized by quantifying the proportional area of each subtype (Fig. 1). For cats with multiple tumors, the average proportion of each histological subtype per animal was calculated using the formula provided in Fig. 1. When 2 subtypes were found in equal proportions (1:1 ratio) within a mass, the subtype associated with a poorer clinical outcome was designated as the predominant subtype. Prognostic ranking was determined using available literature on FMCs,^21^ supplemented by relevant data on canine mammary carcinoma reported by Rasotto et al^19^ when feline-specific data were insufficient.

**Figure 1.**
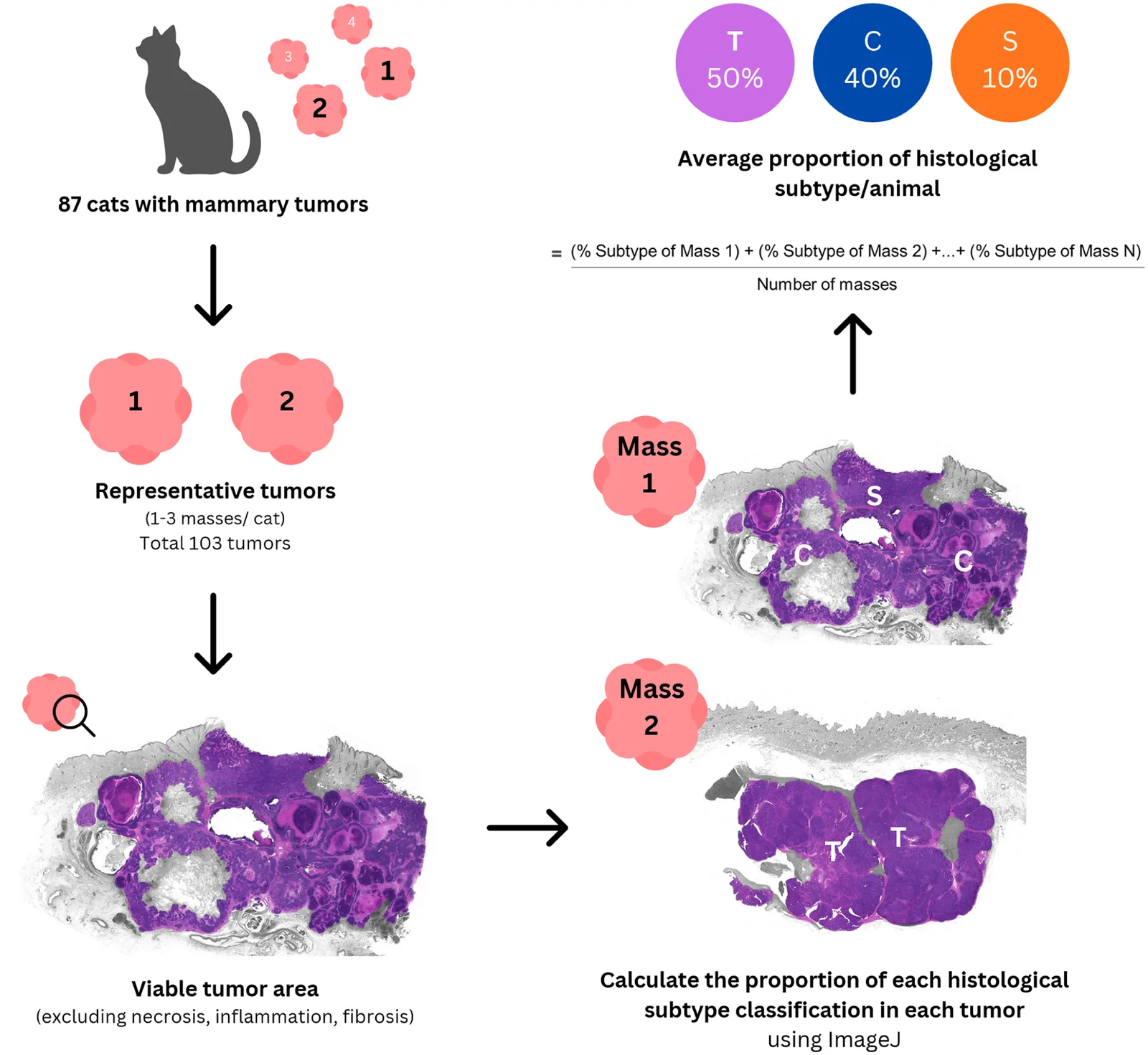
Workflow for determining histological subtype and calculating the average proportion of each histological subtype in feline mammary carcinomas. The study population comprised 87 cats with mammary carcinoma. A representative cohort of 103 tumors from the 87 cats was selected for analysis based on surgical submission. Nonmalignant components (ie, necrosis, fibrosis, and inflammation) were manually outlined and excluded to define the 100% viable tumor area for measurement. Subsequently, each distinct histological subtype population was manually delineated and measured within the viable tumor area, and its proportional area was quantified using ImageJ software. Finally, the average proportion of each histological subtype per animal was calculated using the formula shown. C, comedocarcinoma; S, solid carcinoma; T, tubular carcinoma.

### Definition of Histological Group

Each FMC case was categorized into 1 of 3 histological groups based on the average proportional area of tubular carcinoma observed. This methodology was adopted from the World Health Organization guidelines for histological classification of breast cancer and is consistent with previous studies on human breast cancer.^32,36^ PTC was defined as >90% tubule formation when measured across the total viable tumor area. TMC are tumors that contain 50%–90% tubular carcinoma mixed with other types of mammary carcinomas. NTC (newly defined in this study) includes tumors comprising <50% of tubular structures and predominantly other carcinoma subtypes.

### Immunohistochemistry and Silver Staining

Immunohistochemical Ki-67 labeling was performed on all mammary carcinoma cases following previously published protocols.^6^ Briefly, heat-induced antigen retrieval was performed in sodium citrate buffer (pH 6.0) for 10 minutes. Subsequently, samples were treated with Ki-67 antibody (MIB-1, ready-to-use, Dakocytomation, Glostrup, Denmark) as the primary antibody overnight at 4°C. Poly-horseradish peroxidase anti-mouse/rabbit immunoglobulin G (Envision K5007ENV, Dakocytomation) was applied at 37°C for 30 minutes as a secondary antibody. 3,3′-diaminobenzidine (Envision K5007DAB, Dakocytomation) was applied to induce a chromogenic reaction, followed by counterstaining with hematoxylin. For each case, the Ki-67 index was evaluated in areas with the highest immunohistochemical labeling density. The index was expressed as the percentage of nuclear immunopositivity, as calculated by counting the number of neoplastic cells with nuclear labeling per 1000 cells (field diameter, 0.55 mm; 10 HPFs, 2.37 mm^2^; 400× magnification).^6^ Mammary carcinoma tissue sections with nuclear labeling were used as positive controls for Ki-67. Negative controls were established by replacing the primary antibody with homologous nonimmune sera.

Special staining for argyrophilic nucleolar organizer region (AgNOR) was performed as described previously.^5^ AgNOR analysis was performed using digitized images analyzed on ImageJ software (National Institutes of Health). First, the pathologist captured representative images of the selected high-density areas. Using the cell counter function in ImageJ, discrete brown-black AgNOR dots in 100 positive neoplastic nuclei to obtain the mean ± standard deviation (SD) number of AgNOR dots per cell.

### Definition of Endpoint

Clinical postmastectomy data with at least 2 years of follow-up, including information on locoregional recurrences, lung metastases, and overall survival (OS), were collected retrospectively from medical records. Locoregional recurrence was defined as the presence of neoplastic cells at the surgical site, as confirmed by cytology or histology. Suspected distant metastasis was determined based on thoracic radiography and abdominal ultrasound. OS was defined as the time from mastectomy to death from any cause. Survival data were obtained through medical records and telephone interviews with the owners. Cats that survived at the end of the study or whose owners were lost to follow-up were censored from the survival analysis.

### Statistical Analysis

IBM SPSS 28.0 for Windows (SPSS Inc., Chicago, Illinois) was used for statistical analyses. Continuous variables were assessed for normality using the Kolmogorov-Smirnov test. Ki-67 values, as parametric variables, were expressed as mean ± SD and subcategorized based on the mean value. AgNOR, age, and tumor size were nonparametric variables and expressed as median (interquartile range). AgNOR and age were subcategorized based on their median values. Tumor size was converted into a categorical variable based on the World Health Organization stage criteria,^7^ using a threshold of 3 cm (≤3 cm, >3 cm). Clinicopathological parameters (eg, age, tumor size, surgical margin, clinical stage, and histological grade) were compared among the 3 histological groups using Fisher’s exact test or the chi-square test, with statistical significance set at *P* < .05. Parameters with missing data were recorded as such and omitted from the respective analysis.

Survival analyses were performed using 2 grouping models: model 1 compared the 3 histological groups (PTC, TMC, and NTC), while model 2 combined PTC and TMC into a single tubular carcinoma-based (TC-based) group and compared with the NTC group. The median OS for each histological subtype and grouping model was calculated using the Kaplan-Meier method. Differences between groups were analyzed by log-rank test, with post hoc pairwise comparisons between groups performed using Mantel-Cox analysis. Three histological subtypes (solid carcinoma, micropapillary carcinoma, and ductal carcinoma) were excluded from survival analyses because of their small sample sizes.

Univariable Cox proportional hazards analysis was used to identify and compare variables that were associated with OS. The results were expressed as hazard ratios (HRs) and 95% confidence intervals (CIs). Variables with *P* < .05 were further analyzed using a multivariable model in a backward stepwise manner to determine the strongest risk factors associated with all-cause mortality. Outcomes were considered significant when *P* < .05.

## Results

### Demographic and Clinical Data

This retrospective cohort included 103 tumors from 87 cats diagnosed with FMC. Of these, 73 cats (84%) presented with a single tumor, while 14 cats (16%) had multiple tumors (12 cats with 2 tumors and 2 cats with 3 tumors). The median age of the cats was 10 years (range: 3–20 years). The cohort consisted of 71 (82%) domestic shorthairs and 16/87 (18%) purebred cats: 10 Persian, 4 Siamese, and 2 American shorthair. Reproductive status data were available for 83 cats. Of these, 37 (45%) were spayed before FMC diagnosis, while 46 (55%) remained intact. The reproductive status of the remaining 4 cats was unknown. Furthermore, the specific age or date of the ovariohysterectomy was not available from the records.

The majority of the subjects underwent unilateral mastectomy (41/87; 47%), followed by regional mastectomy (25/87; 29%) bilateral mastectomy (12/87; 14%), simple mastectomy (3/87; 3%), and lumpectomy (3/87; 3%). The surgical type was unknown for 3/87 cats (3%). Medical records showed that 71 (81.6%) cats were treated solely by surgical removal. Eleven cats (12.6%) received adjuvant treatment following mastectomy, including chlorambucil (*n* = 4), doxorubicin (*n* = 4), cyclophosphamide (*n* = 1), metronomic chemotherapy (*n* = 1), and a tyrosine kinase inhibitor (*n* = 1). Treatment information was unknown for 5 (5.7%) cases due to loss to follow-up.

### Tumor Characteristics

Macroscopically, the median tumor diameter was 3.5 cm (interquartile range: 2–5 cm), with sizes ranging from 5 mm to 20 cm. The clinicopathological characteristics of the 87 FMC cases are summarized in Table 1. Although data were available for all cats, specific variables had incomplete information. For example, lymph node status was available only for 45 cats, surgical margin status was available for 85 cats, and anatomical location was available for 86 cats.

**Table 1.** Characteristics of mammary carcinoma specimens.

Clinicopathological Prognostic Variable	*n* (%)
Number of lesions (*n* = 87)	
Single	18 (21%)
Multiple	69 (79%)
Anatomical location (*n* = 86)	
Thoracic	11 (13%)
Abdomen	31 (36%)
Combined	44 (51)
Tumor size (*n* = 87)	
≤3 cm	38 (44%)
>3 cm	49 (56%)
Surgical margin (*n* = 85)	
Complete	21 (25%)
Incomplete	64 (75%)
Lymph node metastasis (*n* = 45)	
Absent	21 (47%)
Present	24 (53%)
Clinical stage (*n* = 87)	
Stage I	5 (6%)
Stage II	20 (23%)
Stage III	53 (61%)
Stage IV	9 (10%)
Lymphovascular invasion (*n* = 87)	
Negative	53 (61%)
Positive	34 (59%)
Grade (Mill et al^14^) (*n* = 87)	
Grade 1	10 (11.5%)
Grade 2	37 (42.5%)
Grade 3	40 (46%)

### Histopathological Subtype

The individual histological subtypes and histological groups are shown in Supplemental Table S1. Tubular carcinoma was the most frequent histological subtype (48/87, 55%; Fig. 2a), followed by comedocarcinoma (17/87, 20%; Fig. 2b), adenosquamous carcinoma (13/87, 15%; Fig. 2c), solid carcinoma (4/87, 5%; Fig. 2d), ductal carcinoma (4/87, 5%; Fig. 2e), and micropapillary carcinoma (1/87; 1%; Fig. 2f). Notably, 2 cases (cases 23 and 37) presented with equivalent ductal and tubular carcinoma components. Consistent with the principle of classifying tumors based on the subtype associated with the poorer prognosis, the 2 cases were thus classified as ductal carcinoma. This is supported by findings in dogs, where ductal-associated carcinoma showed significantly shorter OS (19.8 months) than tubular carcinoma (29.1 months). The distribution of predominant histological subtypes among FMC histological grades are presented in Table 2.

**Figure 2.**
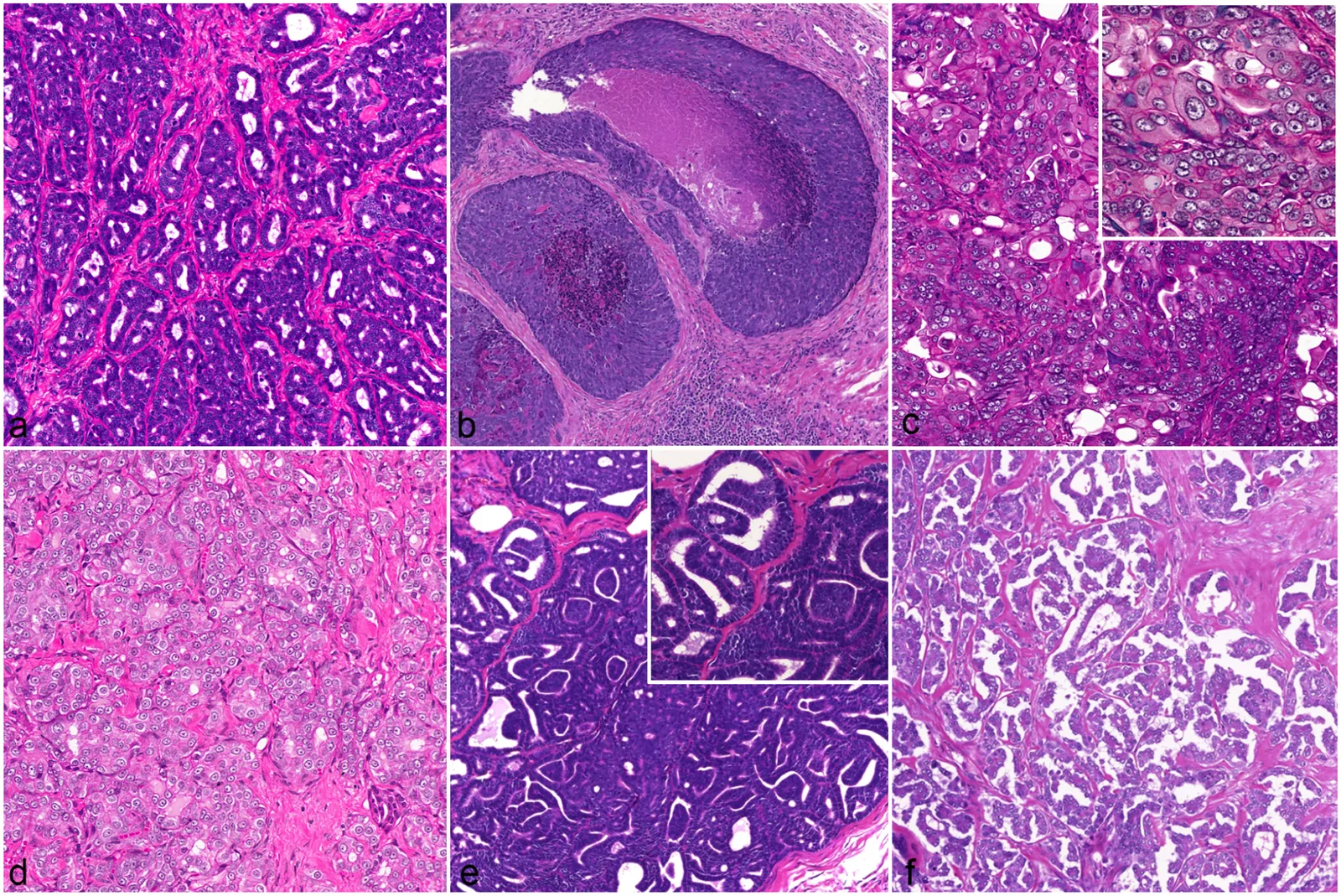
Histological subtypes of feline mammary carcinomas. Hematoxylin and eosin. (a) Tubular carcinoma. Neoplastic epithelial cells predominantly form tubular structures. (b) Comedocarcinoma. Neoplastic epithelial cells form small packets and tubules/acini within neoplastic lobules that are separated by fibrous connective tissue and contain central areas of necrosis. (c) Adenosquamous carcinoma. Neoplastic epithelial cells present as solid sheets or tubules/acini and feature multiple foci of malignant squamous epithelial differentiation. Areas of squamous differentiation comprise at least 25% of the overall neoplastic population. Inset: higher magnification of the neoplastic cells demonstrating marked nuclear pleomorphism and vesiculated nuclei. (d) Solid carcinoma. Neoplastic epithelial cells form solid lobules with minimal to absent tubular differentiation. (e) Ductal carcinoma. Inset: higher magnification of neoplastic epithelial cells forming tubules lined by a double cell layer. (f) Micropapillary carcinoma. Neoplastic cells are arranged in small irregular clusters and small papillae, lack a prominent fibrovascular core, and are surrounded by open lacunar spaces.

**Table 2.** Distribution of histological subtypes across histological grades.

Histological Subtypes (Predominant Subtype^a^)	Grade 1	Grade 2	Grade 3
Tubular carcinoma	8/48 (17%)	21/48 (44%)	19/48 (40%)
Comedocarcinoma	1/17 (6%)	11/17 (65%)	5/17 (29%)
Solid carcinoma	0/4 (0%)	0/4 (0%)	4/4 (100%)
Adenosquamous carcinoma	0/13 (0%)	3/13 (23%)	10/13 (77%)
Ductal carcinoma	1/4 (25%)	2/4 (50%)	1/4 (25%)
Micropapillary carcinoma	0/1 (0%)	0/1 (0%)	1/1 (100%)
Total	10/87 (12%)	37/87 (42%)	40/87 (46%)

a The histological subtype was defined as the predominant morphologic pattern comprising ≥50% of the total tumor area, according to the international histological classifications for feline mammary tumors.^34^

All cases of solid carcinoma and micropapillary carcinoma, together with 77% (10/13) of adenosquamous carcinoma, were classified as grade 3. Most tubular carcinomas (21/48; 44%), comedocarcinomas (11/17; 65%), and ductal carcinomas (2/4; 50%) were categorized as grade 2. Only 10 (11%) cases were classified as grade 1, consisting of ductal carcinomas (1/4; 25%), tubular carcinomas (8/48; 17%), and comedocarcinomas (1/17; 6%).

Of the 87 cats in the study, 37 (43%) exhibited multiple histological subtypes, including intertumoral variation (10/87; 11%) and intratumoral variation (27/87; 31%). The multiple combinations included comedocarcinoma-tubular, adenosquamous-comedocarcinoma, ductal-tubular, and solid-comedocarcinoma-tubular. To further elucidate the potential impact of multiple histological subtypes on the prognosis of FMCs, the histological subtypes were regrouped into 3 groups based on the presence of tubular carcinoma. Of the 87 cats, 34 (39%) had PTC, 16 (18%) had TMC, and 37 (43%) had NTC. The frequency of each histological subtype in these groups is listed in Table 3. In the TMC group, tubular carcinomas often coexisted with comedocarcinomas (11/16; 69%), ductal carcinomas (7/16; 44%), and solid carcinomas (6/16; 38%). The NTC group included cases with single or multiple histological subtypes with <50% tubule formation. Most subtypes in the NTC group included comedocarcinomas (21/37; 57%), adenosquamous carcinomas (14/37; 38%), solid carcinomas (8/37; 22%), ductal carcinomas (5/37; 14%), and micropapillary carcinomas (1/37; 3%). Notably, tubular carcinomas consistently concurred with other histological subtypes in 10/37 specimens (27%) in the NTC group, and the proportion never exceeded 50% of the entire tumor.

**Table 3.** Frequency of each histological subtype across the 3 tubular carcinoma groups.

Histological Subtypes	Groups		
	PTC^a^ (*n* = 34)	TMC^b^ (*n* = 16)	NTC^c^ (*n* = 37)
Tubular carcinoma	34/34 (100%)	16/16 (100%)	10/37 (27%)
Comedocarcinoma	0/16 (0%)	11/16 (69%)	21/37 (57%)
Solid carcinoma	0/16 (0%)	6/16 (38%)	8/37 (22%)
Adenosquamous carcinoma	0/16 (0%)	0/16 (0%)	14/37 (38%)
Ductal carcinoma	0/16 (0%)	7/16 (44%)	5/37 (14%)
Micropapillary carcinoma	0/16 (0%)	0/16 (0%)	1/37 (3%)

Abbreviations: NTC, nontubular carcinomas; PTC, pure tubular carcinomas; TMC, tubular carcinoma mixed with other subtypes.

a PTC: more than 90% tubule formation of the total viable tumor area per cat. ^b^TMC: 50%–90% tubular carcinoma mixed with other subtypes. ^c^NTC: <50% tubular structures, predominantly other carcinoma subtypes.

### Ki-67 and AgNORs

The mean ± SD Ki-67 index was 35% ± 15% (Supplemental Fig. S1). The median AgNOR count was 2.53 dots/cell (interquartile range: 2.0–2.9 dots/cell; Supplemental Fig. S2).

### Comparison of the Clinicopathological Features of the Histological Groups

The clinicopathological features of the histological groups are summarized in Table 4. The NTC group tended to have larger tumors (>3 cm, *P* = .09) with more frequent LVI than the PTC and TMC groups (*P* = .33). The histological group was significantly associated with the clinical stage (*P* = .03). Stage I–II cases were more common in the PTC (7/34; 21%) and TMC (4/16; 25%) groups, whereas they were less frequent in the NTC group (1/37; 3%). Most of the NTC subtype cases were stage III FMCs (32/37; 86%). The proportion of stage IV tumors was smaller across all groups, with the highest percentage observed in the TMC group (3/16; 19%).

**Table 4.** Clinicopathological data on the 3 groups of feline mammary carcinomas.

Parameters	Group, *N* (%)			
	PTC	TMC	NTC	*P* value
	(*n* = 34)	(*n* = 16)	(*n* = 37)	
Age				
≤10 years	19 (56)	7 (44)	19 (51)	.75^a^
>10 years	15 (44)	9 (56)	18 (49)	
Tumor size				
≤3 cm	17 (50)	10 (63)	12 (32)	.09^a^
>3 cm	17 (50)	6 (37)	25 (68)	
Breed				.28^a^
DSH	25 (74)	11 (69)	32 (86)	
Purebred	9 (26)	5 (31)	5 (14)	
Reproductive status				.49^b^
Intact	13 (38)	8 (50)	16 (43)	
Spayed	21 (62)	6 (38)	19 (51)	
Unknown	0	2 (12)	2 (5)	
Surgical margin				.08^a^
Complete	5 (15)	7 (44)	9 (25)	
Incomplete	28 (85)	9 (56)	27 (75)	
Lymph node metastasis				.92^a^
Absent	13 (38)	7 (44)	14 (38)	
Present	21 (62)	9 (56)	23 (62)	
Clinical stage				.**03**^b^
Stage I, II	7 (21)	4 (25)	1 (3)	
Stage III	25 (73)	9 (56)	32 (86)	
Stage IV	2 (6)	3 (19)	4 (11)	
Lymphovascular invasion				.33^a^
Negative	16 (47)	7 (44)	11 (30)	
Positive	18 (53)	9 (56)	26 (70)	
Mitotic count^c^				.14^b^
≤62 mitoses/10 HPFs	31 (91)	11 (69)	31 (84)	
>62 mitoses/10 HPFs	3 (9)	5 (31)	6 (16)	
Grade (Mill et al^14^)				.19^b^
Grade 1	7 (21)	1 (6)	2 (5)	
Grade 2	16 (47)	6 (38)	15 (41)	
Grade 3	11 (32)	9 (56)	20 (54)	
Recurrence				.11^b^
Absent	11 (32)	10 (63)	16 (43)	
Present	21 (62)	5 (31)	15 (41)	
Unknown	2 (6)	1 (6)	6 (16)	
Metastasis				.83^b^
Absent	20 (59)	10 (63)	17 (46)	
Present	12 (35)	5 (31)	15 (41)	
Unknown	2 (6)	1 (6)	5 (13)	
Ki-67				
≤35%	21 (62)	9 (56)	17 (46)	.42^a^
>35%	13 (38)	7 (44)	20 (54)	
AgNOR				
≤2.53 dots/cell	22 (65)	9 (56)	12 (32)	.**02**^a^
>2.53 dots/cell	12 (35)	7 (44)	25 (68)	

Abbreviations: AgNOR, argyrophilic nucleolar organizer region; DSH, domestic shorthair; HPFs, high-power fields; IQR, interquartile range; NTC, nontubular carcinomas; PTC, pure tubular carcinomas; TMC, tubular carcinoma mixed with other subtypes.

a Chi-square test. ^b^Fisher’s exact test. ^c^Cumulative number of mitoses in 10 consecutive fields in the most mitotically active area with a microscope field diameter of 0.55 mm and total area of 2.37 mm^2^ (400× magnification).

While the histological groups did not differ significantly in histological grade, the PTC group had the highest proportions of grade 1 (7/34; 21%) and grade 2 (16/34; 47%) tumors. In contrast, the NTC group had the highest prevalence of grade 3 tumors (20/37; 54%), with only 5% (2/37) being grade 1. The TMC group also frequently presented with grade 3 tumors (9/16; 56%), followed by grade 2 (6/16; 38%) and grade 1 (1/16; 6%) tumors (*P* = .19).

Recurrence more likely occurred in the PTC group than in the TMC and NTC groups (*P* = .11). However, the 3 groups showed no significant difference in metastasis incidence (*P* = .83).

Regarding proliferative markers, NTC neoplasms tended to have higher AgNOR values (>2.53 dots/cell, *P* = .02) and Ki67 indices (>35%) than PTC and TMC tumors, although the Ki-67 index did not differ significantly among the subtypes (*P* = .42).

### Survival Analysis

Follow-up data were available for 76/87 cases; the remaining 11 cases were lost to follow-up and censored from the survival analysis. At the study endpoint (after 2-year postoperatively), 68 cats had died; the 6 surviving cats were also censored cases. A total of 76 cases were included in the survival analysis. For the analysis of histological subtypes, 7 cases (3 ductal, 3 solid, and 1 micropapillary carcinomas) were excluded due to their small sample sizes, leaving 69 cases. Multivariable analysis of OS was conducted on 74 cases, as 2 cases were missing surgical margin data.

The median OS for the cohort was 194 days postoperatively (95% CI: 167–221). Data on local recurrence and metastasis are shown in Table 5. Local recurrence was observed in 41/78 (53%) cats, with the highest recurrence rate occurring in cats with micropapillary carcinoma. Lung metastasis was suspected in 32/79 (40%) of the cases, based on imaging results, with the highest rate observed in ductal carcinomas. No lung metastasis were associated with solid and micropapillary carcinomas.

**Table 5.** Frequency of lymphovascular invasion, recurrence, and lung metastasis in each histological subtype.

Histological Subtypes	Lymphovascular Invasion	Recurrence	Lung Metastasis
Tubular carcinoma	26/48 (54%)	24/ 48 (50%)	16/48 (33%)
Comedocarcinoma	10/17 (59%)	7/17 (41%)	8/17 (47%)
Solid carcinoma	4/4 (100%)	2/4 (50%)	0/4 (0%)
Adenosquamous carcinoma	11/13 (85%)	4/13 (31%)	6/13 (46%)
Ductal carcinoma	1/4 (25%)	3/4 (75%)	2/4 (50%)
Micropapillary carcinoma	1/1 (100%)	1/1 (100%)	0/1 (0%)
Total	53/87 (61%)	41/87 (47%)	32/87 (37%)

Survival was analyzed according to 3 histological classifications: by individual subtype, by histological group model 1 (PTC, TMC, and NTC groups), and by histological group model 2 (TC-based and NTC groups). In model 2, the PTC and TMC groups were combined into a single TC-based group to facilitate a robust comparison between tumors with and without a tubular component. For histological subtype, 69 cases were included in the Kaplan-Meier analysis, which revealed a significant association between histological subtype and OS (*P* < .001, log-rank test). Cats with adenosquamous carcinoma had a significantly shorter OS (60 days, 95% CI: 18–102; *n* = 9) than those with comedocarcinoma (147 days, 95% CI: 33–261; *n* = 16; log-rank test, *P* = .008) and tubular carcinoma (240 days, 95% CI: 189–291; *n* =44; log-rank test, *P* < .001; *n* = 44). Cats with comedocarcinoma had a shorter OS than those with tubular carcinoma but this was not significant (log-rank test, *P* = .051; Fig. 3).

**Figure 3.**
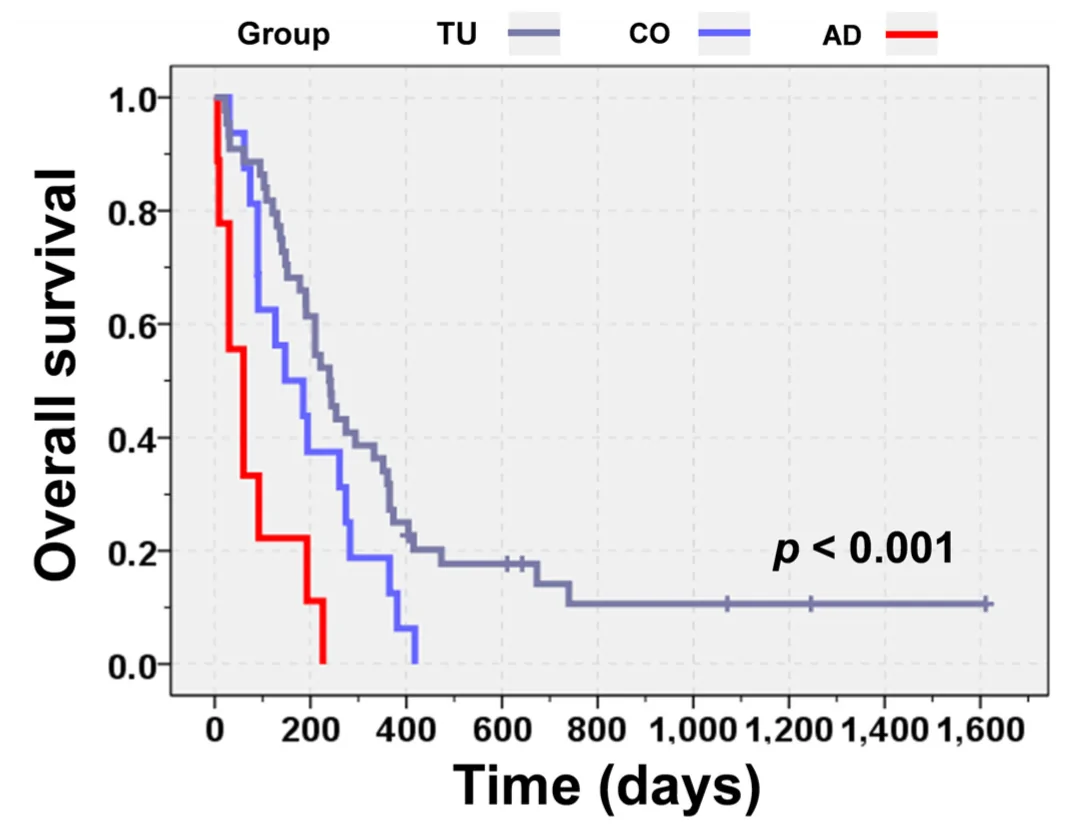
Kaplan-Meier curves depicting a significant difference in overall survival among 69 cats with different predominant subtypes of feline mammary carcinomas (*P* <.001, log-rank test). The median overall survival of tubular (TU) carcinoma, comedocarcinoma (CO), and adenosquamous (AD) were 240 days (95% confidence interval [CI]: 189–291; *n* = 44), 147 days (95% CI: 33–261; *n* = 16), and 60 days (95% CI: 18–102; *n* = 9), respectively. Censored cases are indicated by a vertical dash.

Model 1 was associated with OS of FMCs (*P* = .006, log-rank test; *n* = 76; Fig. 4). The NTC group had the shortest OS at 147 days (95% CI: 44–249; *n* = 31) compared with the PTC group (221 days, 95% CI: 109–333; *n* =31; log-rank test, *P* = .002) and the TMC group (242 days, 95% CI: 180–304; *n* =14; log-rank test, *P* = .049). However, the OS did not differ significantly between the PTC and TMC groups (log-rank test, *P* = .70). Similarly, model 2, which combined the PTC and TMC groups into one TC-based group, revealed that the NTC group had a significantly shorter OS (147 days, 95% CI: 44–250; *n* = 31) than the TC-based group (240 days, 95% CI: 190–290; *n* = 45; log-rank test, *P* = .001; Fig. 5).

**Figure 4.**
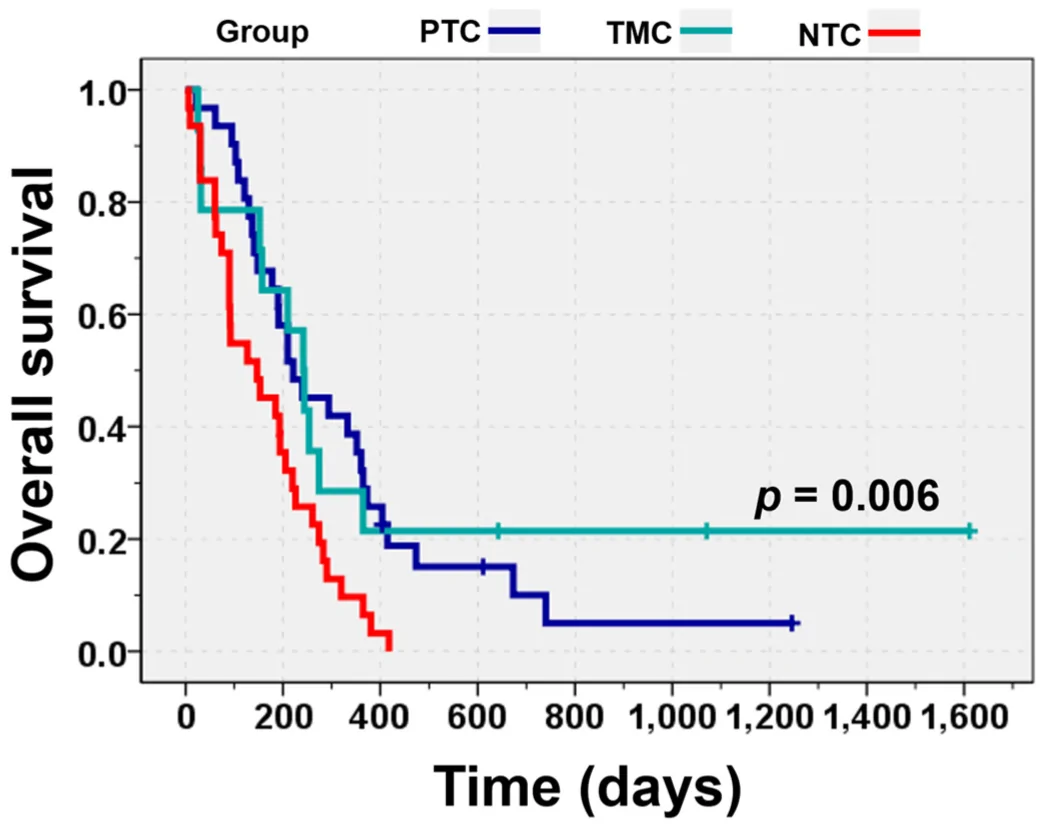
Kaplan-Meier curves showing a significant difference in overall survival among 76 cats stratified according to model 1 histological group for feline mammary carcinomas (*P* = .006, log-rank test). The median overall survival in the pure tubular carcinoma (PTC), tubular and mixed-type carcinoma (TMC), and nontubular carcinoma (NTC) groups were 221 days (95% confidence interval [CI]: 109–333; *n* = 31), 242 days (95% CI: 180–304; *n* = 14), and 147 days (95% CI: 44–249; *n* = 31), respectively. Censored cases are indicated by a vertical dash.

**Figure 5.**
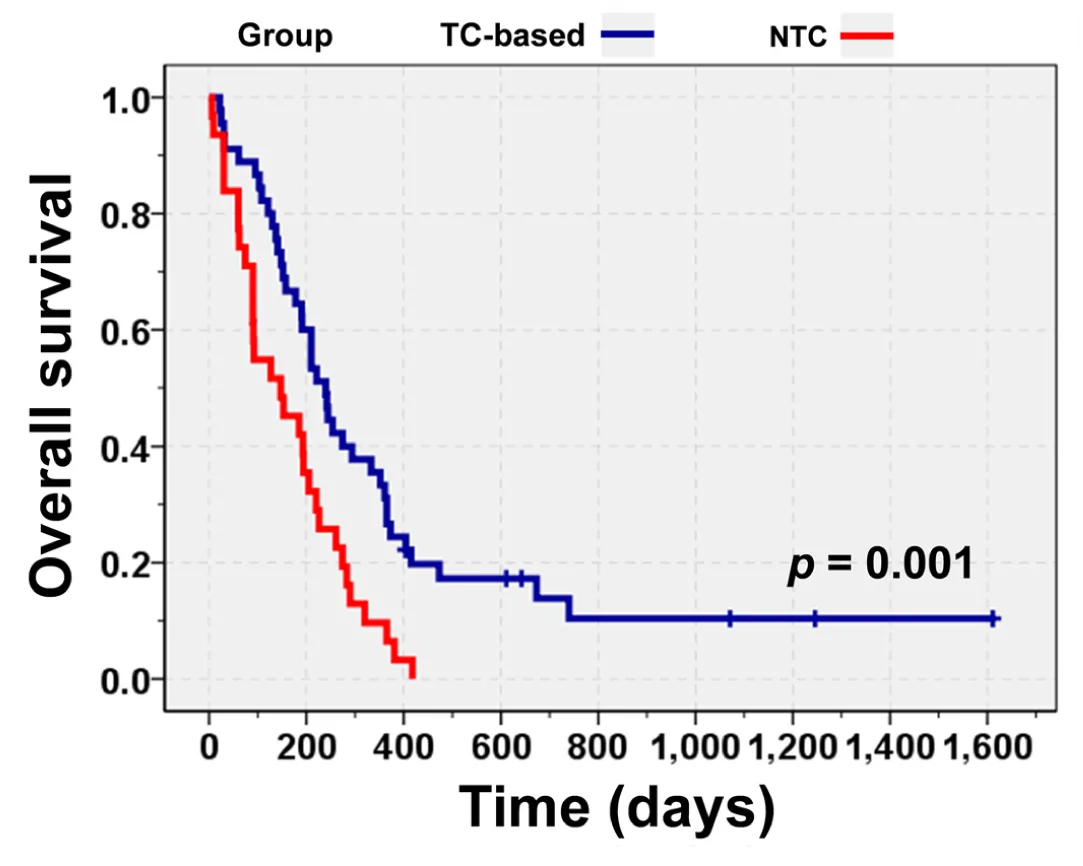
Kaplan-Meier curves demonstrating a significant difference in overall survival of 76 cats stratified according to model 2 histological group for feline mammary carcinomas (*P* = .001, log-rank test). The median overall survival in the tubular carcinoma (TC)-based group (pure tubular carcinoma and tubular and mixed-type carcinoma) was 240 days (95% confidence interval [CI]: 190–290; *n* = 45), while that in the nontubular carcinoma (NTC) group was 147 days postmastectomy (95% CI: 44–250, *n* = 31).

Univariable Cox proportional hazards analysis revealed that all-cause mortality was significantly associated with histological subtype (*P* < .001) and histological group model (model 1, *P* = .006; model 2, *P* = .002; Supplemental Table S2). For individual histological subtypes, adenosquamous carcinoma showed the highest mortality risk (HR: 1.00) compared with comedocarcinoma (HR: 0.32, 95% CI: 0.14–0.76; *P* = .009) and tubular carcinoma (HR: 0.18, 95% CI: 0.08–0.40; *P* < .001).

In model 1, cats in the NTC group had a 2.10-fold higher risk of poor prognosis (95% CI: 1.24–3.55, *P* = .006) than those in the PTC group (HR: 1.00). However, the risk of OS did not differ significantly between the PTC and TMC groups (HR: 0.89, 95% CI: 0.44–1.79; *P* = .74). In model 2, cats in the NTC group showed a 2.12-fold increased risk of poorer OS (95% CI: 1.21–3.54, *P* = .002) than the TC-based group (HR: 1.00).

Other prognostic factors associated with an increased risk of all-cause mortality on univariable analysis included tumor size (>3 cm: HR: 1.86, 95% CI: 1.14–3.03; *P* = .01), incomplete surgical margin (HR: 1.91, 95% CI:1.10–3.32; *P* = .02), advanced-stage FMC (stage III: HR: 3.91, 95% CI: 1.74–8.79, *P* = .001; stage IV: HR: 12.49, 95% CI: 4.37–35.75, *P* < .001), higher FMC grade (grade 2: HR: 2.99, 95% CI: 1.22–7.33, *P* = .02; grade 3: HR: 12.24, 95% CI: 4.49–33.41, *P* < .001), postoperative metastasis (HR: 1.91, 95% CI: 1.17–3.11; *P* = .01), and >2.53 AgNOR dots/cell (HR: 2.74, 95% CI: 1.66–4.51; *P* < .001; Supplemental Table S2).

In the multivariable analysis of OS for 74 cases (2 cases were excluded due to missing surgical margin data), histological classification from model 2 remained a robust prognostic factor. Specifically, the NTC group was associated with a significantly poorer prognosis in FMCs (HR: 1.74, 95% CI: 1.05–2.91, *P* = .03), after adjusting for incomplete surgical margin (HR: 2.45, 95% CI: 1.34–4.49, *P* = .004), advanced-stage FMC (stage III: HR: 2.73, 95% CI:1.15–6.47, *P* = .02; stage IV: HR: 15.10, 95% CI: 4.70–48.55, *P* < .001), or grade 3 FMC (HR: 12.40, 95% CI: 3.89–39.60, *P* < .001; Table 6).

**Table 6.** Multivariable analysis of prognostic factors affecting overall survival in feline mammary carcinomas.

Parameter (N = 74)	Overall survival		*p* value
	HR	95% CI	
Surgical margin			
Complete (n = 21)	1.00	Reference^a^	-
Incomplete (n = 53)	2.45	1.34–4.49	**0.004**
Clinical stage			**<0.001**
Stage I, II (n = 11)	1.00	Reference	**-**
Stage III (n = 54)	2.73	1.15–6.47	**0.02**
Stage IV (n = 9)	15.10	4.70–48.55	**<0.001**
Histological group model 2			
TC-based (n= 44)	1.00	Reference	**–**
NTC (n = 30)	1.74	1.05–2.91	**0.03**
Grade			**<0.001**
Grade 1 (n = 8)	1.00	Reference	**-**
Grade 2 (n = 32)	2.77	1.00–7.71	0.051
Grade 3 (n = 34)	12.40	3.89–39.60	**<0.001**

a This reference group had a hazard ratio (HR) = 1.00. Groups with HR > 1.00 and *P* < 0.05 were associated with shorter overall survival.

Abbreviations: CI, confidence interval; NTC, non-tubular carcinomas; PT, tubular carcinomas; TC, tubular carcinoma; TMC, tubular carcinoma mixed with other subtypes.

## Discussion

This study proposed criteria for classifying multiple FMC histological subtypes into 3 groups based on the presence of tubular carcinoma, adapting the 2012 World Health Organization classification for human breast cancer^32^ to FMCs. Subsequently, the prognostic significance of these groups was evaluated by comparing clinicopathological prognostic factors, proliferative markers, and clinical outcomes. The results indicated that tubular carcinoma, whether pure or mixed with other subtypes, was associated with a more favorable prognosis, whereas NTC tumors exhibited the worst clinical outcomes. Among individual subtypes, adenosquamous carcinoma, characterized by high histological grade and metastatic potential, had the poorest prognosis. Multivariable analysis identified the NTC group, incomplete surgical margin, advanced clinical stage, and high histological grade as significant negative prognostic factors.

In cats, 33.6%–87.5% of mammary carcinomas present as a mixed population of histological subtypes.^16,23,33^ Although different histological subtypes reflect varying morphological patterns, characteristics, and cellular atypia,^9^ their prognostic significance in FMCs remains controversial.^21^ Because no standardized guidelines for classifying FMCs with multiple histological patterns have been established, the influence of minor or coexisting subtypes on patient prognosis remains unclear. Current general practice typically considers only the single most prominent unfavorable subtype, which may underestimate the true prognostic risk due to morphological heterogeneity within the tumor.^6,14,27^ To address this gap, we adopted criteria used in human breast cancer for grouping multiple histological patterns based on the presence of tubular carcinoma. The issue of tubular and mixed-type carcinoma is well addressed by specific criteria in the 2012 World Health Organization classification for human breast cancer.^32^ Adapting this comparative oncology framework in veterinary medicine is highly useful for improving prognostic accuracy and optimizing therapeutic strategies.

Feline and canine mammary carcinomas exhibit comparable histological features across subtypes.^3^ In dogs, survival analysis and detailed studies on disease progression, including rates of recurrence and metastasis for various histological subtypes, have been established in large cohorts.^19,26^ In this study, canine data were used as useful comparative evidence where feline-specific prognostic data were insufficient; however, interspecies biological differences may limit the direct applicability of this information to FMC.

Tubular carcinoma was the most common subtype observed in the present study (55%), which is consistent with previous studies^33,34^ that reported the frequent coexistence of this subtype with comedocarcinoma, solid carcinoma, and ductal carcinoma. Furthermore, when considering histological subtype, tubular carcinoma exhibited more favorable characteristics and significantly longer OS than other subtypes, aligning with previous findings.^14,23,34^ Interestingly, the recurrence rate for tubular carcinoma in FMCs in our study was higher than that reported in dogs, although the metastatic rate was comparable.^19^

Adenosquamous carcinoma, comedocarcinoma, and solid carcinoma were associated with poorer prognosis in dogs.^19,26^ However, data on the prognosis of these subtypes in FMCs are limited. In our study, adenosquamous carcinoma had the poorest prognosis among all analyzed histological subtypes, with the shortest OS of 153 days postoperatively. This subtype was primarily associated with high histological grades and exhibited a high prevalence of LVI, reflecting its aggressive local behavior and high metastatic potential. However, adenosquamous FMCs showed lower recurrence and metastatic rates than their canine counterparts.^19^

In the present study, most comedocarcinomas were grade 2 or 3 and exhibited a trend toward a poorer prognosis compared with tubular carcinomas. Furthermore, comedocarcinomas exhibited a higher recurrence rate in FMCs than in canine ones, although the metastatic rate was lower.^19^ The aggressive nature of comedocarcinoma is further indicated by central necrotic areas, suggesting rapid growth and an increased risk of tumor progression.^20,29^ Given the poorer prognostic trend and more aggressive characteristics of comedocarcinoma and adenosquamous carcinoma, additional treatment strategies may be required following removal of these tumors.

Ductal, solid, and micropapillary carcinomas were rare in our study, excluding them from survival analysis. Among these, ductal carcinoma exhibited a potentially favorable prognosis characterized by less frequent LVI, similar to previous findings in dogs.^9^ In contrast, the aggressive behavior of solid and micropapillary carcinomas was evident, as all cases with these subtypes were classified as high-grade and presented with LVI. These findings aligned with those of prior studies in both canine and feline populations.^4,20,23,24^ However, unlike in previous studies,^19^ the absence of lung metastasis in solid and micropapillary carcinomas was attributable to the small sample size. Despite this limitation, our study observed notably high recurrence rates in micropapillary, ductal, and solid carcinomas. Consequently, further studies involving larger populations are needed to validate the behavior and prognosis of these FMC subtypes.

This study found significant variation in histological subtypes in FMCs, with 42.5% exhibiting multiple histological subtypes, of which 12% involved intertumoral heterogeneity and 31% involved intratumoral heterogeneity. These findings support the morphological heterogeneity in FMCs reported by Mohamed Ali et al.^16^ Given the heterogeneity in FMCs, we proposed the new approach of categorizing the different histological subtypes into 3 groups—PTC, TMC, and NTC—based on the presence of tubular carcinoma, as adopted from the 2012 World Health Organization guidelines for the histological classification of breast cancer.^32^

The PTC group exhibited the most favorable prognosis among the 3, mirroring the behavior of tubular carcinoma reported in humans and dogs.^9,14,18,19,31,36^ This was further confirmed by its lower proliferative activity and significant longer OS than the NTC group. Despite these favorable characteristics, the PTC group showed the highest tumor recurrence rate, which may be attributed to a higher proportion of incomplete resection margins.

The NTC group exhibited the poorest prognosis and was associated with the shortest OS compared with the TC-based groups. This may be because most NTC cases were in advanced stages and exhibited higher histologic grades, AgNOR values, prevalence of lymph node metastasis, and LVI. Furthermore, the latter 2 factors indicate a higher likelihood of metastasis in NTC tumors, which may be potentially driven by inherently aggressive subtypes, particularly adenosquamous, solid, and micropapillary carcinoma.^4,24^

The TMC group demonstrated a mixture of tubular carcinoma and other subtypes, particularly comedocarcinoma, ductal carcinoma, and solid carcinoma, similar to previous reports.^16,33^ TMC tumors also varied in their biological behaviors. Although they were more frequently in advanced clinical stages and with high histological grades, the TMC group demonstrated the longest OS among the groups. Recurrence and metastasis were less common in this group, which may be associated with smaller tumor sizes and complete surgical margins, which contributed to improved outcomes in the group. In contrast, human breast cancer with a TMC component presents differently. Tubular carcinoma in humans is often concurrent with ductal carcinoma in situ.^31,36^ Several studies in humans reported a poorer prognosis for breast cancer with TMC, as human TMC tumors tend to have larger tumor sizes and greater lymph node metastasis. However, long-term outcomes (eg, recurrence, metastasis, and survival rates) did not differ from the PTC subtype.^31,36^ These contrasting findings indicate that although the presence of tubular carcinoma may positively impact prognosis in feline cases, the additional invasive components found in both felines and humans complicate the outcomes. The parallels between humans and cats highlight the need for further investigations involving larger populations and molecular studies in FMCs with multiple subtypes to improve accuracy in tumor classification and to elucidate the underlying disease mechanisms.

Multivariable analysis identified an incomplete surgical margin, advanced clinical stage, and high histological grade as variables related to poor prognosis in FMCs, in agreement with previous studies.^3,4,7,14,23,35^ The univariable analysis of proliferative markers revealed that only AgNOR counts were significantly correlated with FMC prognosis. High AgNOR counts were frequently observed in the NTC group, indicating aggressive disease. Our findings support AgNOR as a potentially informative prognostic marker in this cohort, consistent with prior reports in humans, dogs, and cats.^2,5,12,17,22^ However, its comparative utility relative to Ki-67 remains uncertain. Variations in study design, geographic topology, immunohistochemistry protocols, and tumor heterogeneity likely influence cutoff values of AgNOR and Ki-67; therefore, our observed thresholds should be considered an exploratory value specific to this dataset rather than a standardized diagnostic cutoff for FMCs. Further independent studies using standardized methods and cutoffs are required to establish the utility of these markers in routine prognostic prediction in FMCs.

The inherent retrospective nature of this study presents several key limitations. Incomplete clinical data in medical records and the varying number of tumor specimens per cat affect the completeness of the parameters analyzed. Outcome differences may also reflect confounding by treatment allocation, stage, surgical margin status, and clinical decision-making in a retrospective setting. Furthermore, the retrospective design introduced a potential selection bias, which may limit the generalizability of our findings. Specifically, the assignment of equal-proportion cases based on subtypes with poorer clinical behavior may have introduced classification bias and should be interpreted with caution. The lack of an interobserver agreement analysis for histological classification limit the reliability of our methodology, and a small sample size significantly impacts the robustness of the statistical comparisons. In particular, the small sample size restricted the inferential analysis for rare histological subtypes, thereby limiting the definitive prognostic conclusions that can be drawn or the optimal cutoffs that can be determined for proliferative markers for these specific subgroups. Further validation of the prognostic value of these multiple subtype carcinomas should be conducted in a larger, prospective sample size.

In conclusion, this study addressed the diagnostic challenges posed by histological heterogeneity in FMCs by establishing a set of grouping criteria adopted from a human breast cancer classification scheme and based on the presence of tubular carcinoma. We found that NTCs had the poorest prognosis due to the inclusion of aggressive subtypes, such as adenosquamous carcinoma, whereas TCs demonstrated more favorable outcomes. This comparative oncology approach provides a potentially useful framework that indicates the significant prognostic value of histological classification. However, this study was limited by its retrospective design and the inherent lack of robust inferential analysis of rare histological subtypes due to incomplete data. These findings should be considered exploratory and hypothesis-generating pending validation in an independent and prospective cohort. Future studies should evaluate the histological grouping schemes proposed here and advance the standardized classification of multiple histological subtypes in FMCs by incorporating molecular analyses to enhance prognostic accuracy and optimize treatment strategies.

## Supplemental Material

sj-pdf-1-vet-10.1177_03009858261462564 – Supplemental material for Clinicopathological characteristics and clinical outcomes of multiple histological subtypes in feline mammary carcinomas Supplemental material, sj-pdf-1-vet-10.1177_03009858261462564 for Clinicopathological characteristics and clinical outcomes of multiple histological subtypes in feline mammary carcinomas by Pruettha Aruvornlop, Nlin Arya, Sirintra Sirivisoot, Tanit Kasantikul, Rassameepen Phonarknguen and Walasinee Sakcamduang in Veterinary Pathology

## Data Availability

The data analyzed in this study are available from the “Supplemental Material” section.
