# Supplementary material for: Clinicopathological characteristics and clinical outcomes of multiple histological subtypes in feline mammary carcinomas
Source: Vet Pathol. 2026 Jul 13;63(6):1025–37. doi: 10.1177/03009858261462564 (PMC13714229; doi:10.1177/03009858261462564)
Supplement: Supplementary file 1 [file sj-pdf-1-vet-10.1177_03009858261462564.pdf]

Supplemental Materials

Clinicopathological characteristics and clinical outcomes of multiple histological subtypes in feline mammary carcinomas

Pruettha Aruvornlop, Nlin Arya, Sirintra Sirivisoot, Tanit Kasantikul, Walasinee Sakcamduang, Rassameepen Phonarknguen

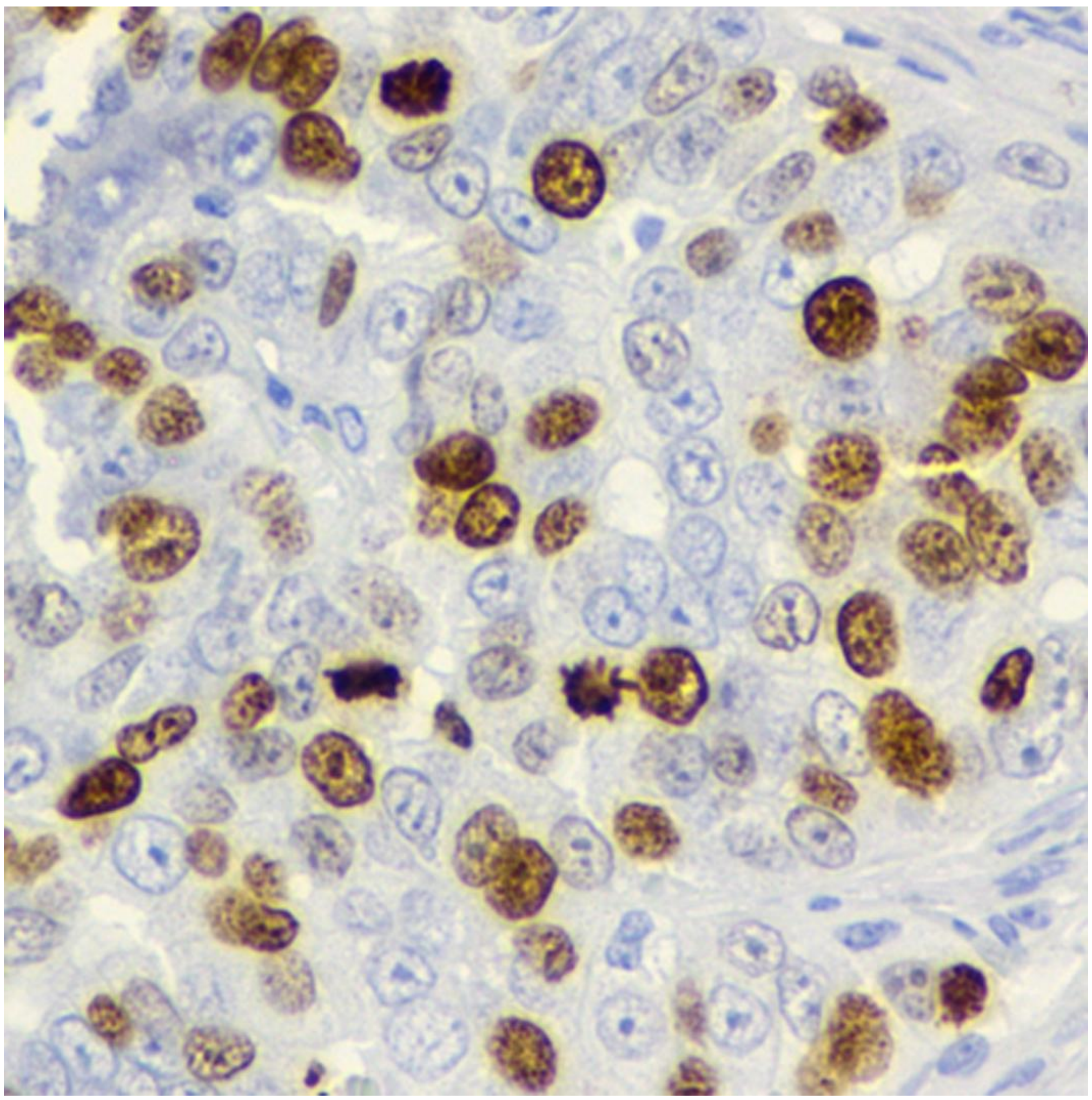

Supplemental Figure S1. Ki-67 immunohistochemistry in a feline mammary comedocarcinoma.

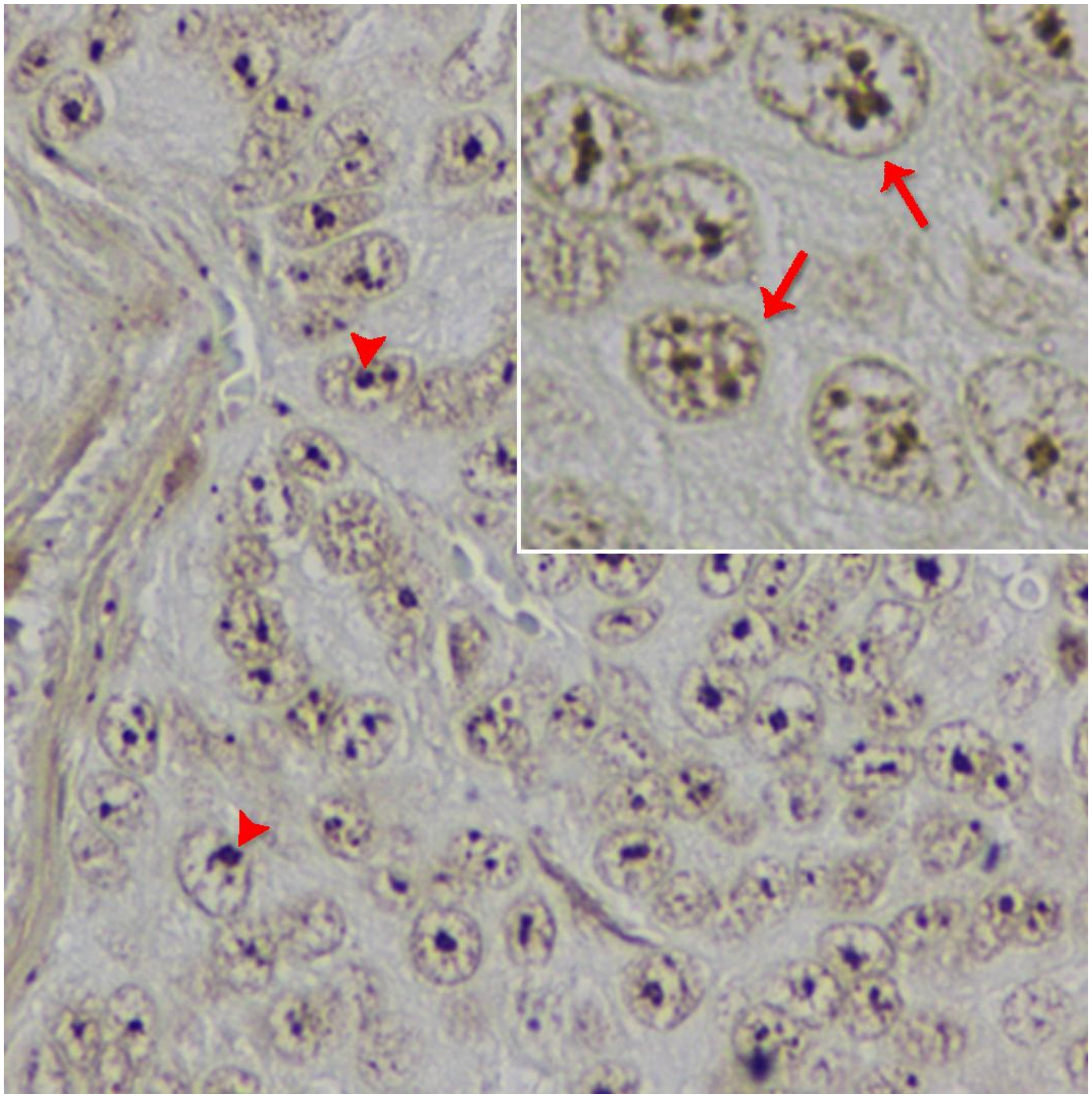

Supplemental Figure S2. Argyrophilic nucleolar organizer region (AgNOR) staining of feline mammary comedocarcinoma tissue. The AgNOR appears as black-brown dots within cell nuclei (arrowheads). Inset: Neoplastic epithelial cells with five or more AgNOR dots/cell (red arrow).

Supplemental Table S1. Histopathological findings in feline mammary carcinomas.

Case no.	Histological subtype (total %)	Prominent subtype	Subgroup	Nuclear form	Mitoses/ 10 HPF	LVI	Grade	Status
1	TU (52), CO (33), DU (15)	TU	TMC	> 5%	41	–	2	death
2	TU (60), SO (25), CO (15)	TU	TMC	> 5%	25	+	3	death
3	TU (67), CO (26), SO (7)	TU	TMC	> 5%	46	+	3	death
4	TU (90), CO (10)	TU	PTC	> 5%	49	+	3	death
5	CO (58), TU (42)	CO	NTC	> 5%	97	+	3	death
6	SO (58), TU (42)	SO	NTC	> 5%	36	+	3	death
7	CO (80), SO (20)	CO	NTC	> 5%	51	–	2	death
8	CO (59), TU (41)	CO	NTC	≤ 5%	65	–	2	death
9	TU (49), CO (47), DU (4)	TU	TMC	> 5%	32	–	2	alive
10	CO (74), TU (26)	CO	NTC	> 5%	51	–	2	death
11	TU (70), SO (16), CO (14)	TU	TMC	> 5%	64	–	3	death
12	TU (90), CO (10)	TU	PTC	≤ 5%	30	–	1	alive
13	TU (73), CO (27)	TU	TMC	> 5%	92	+	3	death
14	AD (50), DU (42), TU (8)	AD	NTC	> 5%	154	+	3	death
15	TU (70), CO (25), SO (5)	TU	TMC	> 5%	68	+	3	death
16	TU (52), CO (48),	TU	TMC	> 5%	14	–	2	alive
17	TU (90), CO (10)	TU	PTC	> 5%	54	–	2	death
18	TU (65), DU (28), SO (7)	TU	TMC	> 5%	51	–	2	death
19	CO (50), TU (30), SO (20)	CO	NTC	> 5%	90	+	3	death
20	AD (66), TU (20), CO (8), DU (	AD	NTC	> 5%	38	+	3	death
21	AD (86), CO (14)	AD	NTC	> 5%	23	+	3	lost to follow-up
22	TU (60), CO (40)	TU	TMC	> 5%	76	+	3	death
23	DU (50), TU (50)	DU	TMC	> 5%	49	+	3	death
24	CO (74), TU (36)	CO	NTC	> 5%	51	+	3	death
25	TU (80), CO (20)	TU	TMC	≤ 5%	57	+	2	death
26	TU (64), SO (36)	TU	TMC	≤ 5%	99	+	3	death
27	AD (50), CO (25), TU (25)	AD	NTC	> 5%	41	+	3	death
28	TU (97), SO (3)	TU	PTC	≤ 5%	63	–	2	death
29	AD (100)	AD	NTC	> 5%	55	+	3	death
30	CO (87), SO (13)	CO	NTC	> 5%	49	+	3	death
31	TU (77), DU (12), CO (11)	TU	TMC	≤ 5%	30	–	1	alive
32	TU (90), CO (10)	TU	PTC	≤ 5%	11	–	1	alive
33	AD (70), DU (30)	AD	NTC	> 5%	56	–	2	lost to follow-up
34	SO (54), CO (20), TU (26)	SO	NTC	> 5%	20	+	3	lost to follow-up
35	TU (87), SO (13)	TU	TMC	> 5%	33	+	3	lost to follow-up
36	TU (90), SO (10)	TU	PTC	> 5%	14	–	2	lost to follow-up
37	DU (50), TU (50)	DU	TMC	> 5%	35	–	2	lost to follow-up
38	CO (89), AD (11)	CO	NTC	> 5%	41	–	2	death
39	TU (100)	TU	PTC	≤ 5%	14	+	2	death
40	CO (100)	CO	NTC	≤ 5%	36	+	2	death
41	CO (100)	CO	NTC	> 5%	49	+	3	death
42	TU (100)	TU	PTC	≤ 5%	40	+	2	death
43	CO (100)	CO	NTC	≤ 5%	35	–	1	death
44	AD (100)	AD	NTC	≤ 5%	56	+	2	death
45	TU (100)	TU	PTC	≤ 5%	41	+	2	death
46	TU (100)	TU	PTC	≤ 5%	44	–	1	death
47	AD (100)	AD	NTC	> 5%	45	+	3	death
48	CO (100)	CO	NTC	≤ 5%	45	+	2	death
49	AD (100)	AD	NTC	> 5%	49	+	3	death

50	CO (100)	CO	NTC	≤ 5%	68	–	2	death
51	TU (100)	TU	PTC	≤ 5%	86	+	3	death
52	SO (100)	SO	NTC	> 5%	59	+	3	death
53	TU (100)	TU	PTC	> 5%	46	–	2	death
54	TU (100)	TU	PTC	≤ 5%	42	–	1	death
55	TU (100)	TU	PTC	> 5%	60	–	2	death
56	CO (100)	CO	NTC	≤ 5%	58	+	2	lost to follow-up
57	TU (100)	TU	PTC	≤ 5%	20	–	1	death
58	DU (100)	DU	NTC	≤ 5%	47	–	1	death
59	SO (100)	SO	NTC	> 5%	22	+	3	death
60	TU (100)	TU	PTC	≤ 5%	32	+	2	death
61	TU (100)	TU	PTC	> 5%	64	+	3	death
62	TU (100)	TU	PTC	> 5%	25	+	3	death
63	CO (100)	CO	NTC	≤ 5%	35	+	2	death
64	AD (100)	AD	NTC	> 5%	15	+	3	death
65	TU (100)	TU	PTC	> 5%	43	–	2	alive
66	TU (100)	TU	PTC	> 5%	37	–	2	death
67	TU (100)	TU	PTC	> 5%	44	+	3	death
68	TU (100)	TU	PTC	≤ 5%	42	+	2	death
69	TU (100)	TU	PTC	> 5%	36	+	3	death
70	AD (100)	AD	NTC	> 5%	43	+	3	death
71	DU (100)	DU	NTC	> 5%	50	–	2	death
72	TU (100)	TU	PTC	≤ 5%	25	+	2	death
73	CO (100)	CO	NTC	≤ 5%	41	+	2	death
74	TU (100)	TU	PTC	> 5%	45	+	3	death
75	TU (100)	TU	PTC	> 5%	36	+	3	death
76	TU (100)	TU	PTC	≤ 5%	25	+	2	death
77	TU (100)	TU	PTC	> 5%	5	–	2	death
78	TU (100)	TU	PTC	> 5%	30	+	3	death
79	TU (100)	TU	PTC	≤ 5%	30	–	1	death
80	CO (100)	CO	NTC	≤ 5%	68	–	2	death
81	TU (100)	TU	PTC	> 5%	37	–	2	death
82	AD (100)	AD	NTC	> 5%	39	+	3	lost to follow-up
83	TU (100)	TU	PTC	> 5%	19	+	3	death
84	TU (100)	TU	PTC	> 5%	12	+	3	lost to follow-up
85	MI (100)	MI	NTC	> 5%	28	+	3	death
86	TU (100)	TU	PTC	≤ 5%	11	–	1	lost to follow-up
87	AD (100)	AD	NTC	> 5%	53	–	2	lost to follow-up

Abbreviations: TU, tubular carcinoma; SO, solid carcinoma; DU, ductal carcinoma; CO, comedocarcinoma; AD, adenosquamous carcinoma; PTC, pure tubular carcinoma; TMC, tubular and invasive carcinomas; NTC, other invasive carcinomas; LVI, lymphovascular invasion.

Supplemental Table S2. Univariable analysis of prognostic factors affecting overall survival in feline mammary carcinomas.

Parameters	N	Overall survival		*P* value
		Hazard ratio	95% CI	
Age (n = 76)				
≤ 10 years	36	1.00	Reference[a]	-
> 10 years	40	1.16	0.72–1.86	0.54
Tumour size (n = 76)				
≤ 3 cm	34	1.00	Reference	**-**
> 3 cm	42	1.86	1.14–3.03	**0.01**
Breed (n = 76)				
DSH	60	1.00	Reference	-
Purebred	16	1.00	0.55–1.80	0.99
Reproductive status (n = 73)				
Intact	36	1.00	Reference	-
Spayed	37	0.91	0.56–1.46	0.68
Number of lesion (n = 76)				
Single	16	1.00	Reference	
Multiple	60	1.36	0.76–2.41	0.30
Anatomical location (n = 75)				0.37
Thoracic	9	1.00	Reference	-
Abdomen	27	1.77	0.80–3.95	0.16
Combined	39	1.47	0.67–3.20	0.34
Treatment (n = 76)				
Surgery only	66	1.00	Reference	-
Surgery and adjuvant chemotherapy	10	0.88	0.43–1.78	0.65
Surgical margin (n = 74)				
Complete	21	1.00	Reference	-
Incomplete	53	1.91	1.10 – 3.32	**0.02**
Lymph node metastasis (n = 45)				
Absent	21	1.00	Reference	-
Present	24	1.79	0.90–3.57	0.1
Clinical stage (n = 76)				**<0.001**
Stage I, II	11	1.00	Reference	-
Stage III	56	3.91	1.74–8.79	**0.001**
Stage IV	9	12.49	4.37–35.75	**<0.001**
Histological subtype[b] (n = 69)				**0.001**
Adenosquamous carcinoma	9	1.00	Reference	**-**
Comedocarcinoma	16	0.32	0.14–0.76	**0.009**
Tubular carcinoma	44	0.18	0.08–0.40	**<0.001**
Ductal carcinoma	3			
Solid carcinoma	3			
Micropapillary carcinoma	1			
Histological subgroup: model1 (n = 76)				**0.01**
PTC	31	1.00	Reference	-
TMC	14	0.89	0.44–1.79	0.74
NTC	31	2.10	1.24–3.55	**0.006**

Histological subgroup: model 2 (n = 76)				
TC-based (PT and TMC)	45	1.00	Reference	**-**
NTC	31	1.47	1.15– 1.88	**0.002**
Lymphovascular invasion (n = 76)				
Negative	29	1.00	Reference	
Positive	47	3.50	2.04–5.99	**<0.001**
Mitotic count[c] (n = 76)				
≤62 mitoses/ 10 HPFs	62	1.00	Reference	**-**
>62 mitoses/ 10 HPFs	14	1.50	0.82–2.72	0.19
Grade (Mill et al., 2015) (n = 76)				**<0.001**
Grade 1	9	1.00	Reference	-
Grade 2	32	2.99	1.22–7.33	**0.02**
Grade 3	35	12.24	4.49–33.41	**<0.001**
Recurrence (n = 76)				
Absent	37	1.00	Reference	-
Present	39	1.37	0.84– 2.22	0.21
Metastasis (n = 76)				
Absent	45	1.00	Reference	-
Present	31	1.91	1.17–3.11	**0.01**
Ki-67 (n = 76)				
≤ 35%	40	1.00	Reference	-
> 35%	36	1.15	0.71–1.84	0.57
AgNOR (n = 76)				
≤ 2.53 dots/cell	37	1.00	Reference	**-**
> 2.53 dots/cell	39	2.74	1.66–4.51	**<0.001**

[a]This reference subgroup had a hazard ratio = 1.00. The subgroups with HR > 1.00 and *P* < 0.05 were associated with shorter overall survival.

[b]Solid carcinoma, ductal carcinoma, and micropapillary carcinoma was excluded from survival analysis due to the small sample size.

[c]Cumulative number of mitoses in 10 consecutive fields in the most mitotically active area with a microscope field diameter of 0.55 mm and total area of 2.37 mm^2 (400× magnification).

Abbreviations: CI, confidence interval; DSH, domestic shorthair; HPFs, high-power fields; NTC, non-tubular carcinoma; PTC, pure tubular carcinoma; TC, tubular carcinoma; TMC, tubular carcinoma mixed with other subtypes
